# An evidence-based definition of anemia for singleton, uncomplicated pregnancies

**DOI:** 10.1371/journal.pone.0262436

**Published:** 2022-01-13

**Authors:** Amanda C. Zofkie, W. Holt Garner, Rachel C. Schell, Alexandra S. Ragsdale, Donald D. McIntire, Scott W. Roberts, Catherine Y. Spong

**Affiliations:** 1 Maternal-Fetal Medicine Division, Department of Obstetrics and Gynecology, University of Texas Southwestern Medical Center, Parkland Health and Hospital System, Dallas, Texas, United States of America; 2 Maternal-Fetal Medicine Division, Department of Obstetrics and Gynecology, Washington University School of Medicine in St. Louis, St. Louis, Missouri, United States of America; Qatar University, QATAR

## Abstract

**Background:**

The definition for anemia in pregnancy is outdated, derived from Scandinavian studies in the 1970’s to 1980’s. To identity women at risk of blood transfusion, a common cause of Severe Maternal Morbidity, a standard definition of anemia in pregnancy in a modern, healthy United States cohort is needed.

**Objective:**

To define anemia in pregnancy in a United States population including a large county vs. private hospital population using uncomplicated patients.

**Materials and methods:**

Inclusion criteria were healthy women with the first prenatal visit before 20 weeks. Exclusion criteria included preterm birth, preeclampsia, hypertension, diabetes, short interval pregnancy (<18 months), multiple gestation, abruption, and fetal demise. All women had iron fortification (Ferrous sulfate 325 mg daily) recommended. The presentation to care and pre-delivery hematocrits were obtained, and the percentiles determined. A total of 2000 patients were included, 1000 from the public county hospital and 1000 from the private hospital. Each cohort had 250 patients in each 2011, 2013, 2015, and 2018. The cohorts were compared for differences in the fifth percentile for each antepartum epoch. Student’s t-test and chi-squared statistical tests were used for analysis, p-value of ≤0.05 was considered significant.

**Results:**

In the public and private populations, 777 and 785 women presented in the first trimester while 223 and 215 presented in the second. The women at the private hospital were more likely to be older, Caucasian race, nulliparous, and present earlier to care. The fifth percentile was compared between the women in the private and public hospitals and were clinically indistinguishable. When combining the cohorts, the fifth percentile for hemoglobin/hematocrit was 11 g/dL/32.8% in the first trimester, 10.3 g/dL/30.6% in the second trimester, and 10.0 g/dL/30.2% pre-delivery.

**Conclusions:**

Fifth percentile determinations were made from a combined cohort of normal, uncomplicated pregnancies to define anemia in pregnancy. Comparison of two different cohorts confirms that the same definition for anemia is appropriate regardless of demographics or patient mix.

## Introduction

Maternal mortality complicated 20.1 per 100,00 live births in 2019 in the United States, a higher rate than other medically developed countries [1]. As a proxy to mortality, Severe Maternal Morbidity (SMM) is a set of risk metrics measured to identify these adverse outcomes, which are often preventable. While the potential morbidities in pregnancy are numerous, the Centers for Disease Control (CDC) has specifically identified the need for blood transfusion as a leading cause of SMM, an adverse outcome which has increased significantly in the last two decades [2, 3]. As a necessary predictor for the risk of transfusion, and those women at risk for evaluation and treatment of anemia, an evidence-based definition of anemia in pregnancy is of paramount importance.

Anemia has been traditionally defined by the CDC and World Health Organization (WHO) as hemoglobin or hematocrit values less than the fifth percentile of the population in pregnant and nonpregnant populations [4, 5]. In pregnancy, this translates to a hemoglobin of less than 11 g/dL or a hematocrit less than 33% in the first and third trimesters and a hemoglobin of less than 10.5 g/dL or hematocrit less than 32% in the second trimester, accounting for the expanded plasma volume of pregnant women [3]. These definitions were originally defined in 1989 and reaffirmed in 1998 [4, 6]. These fifth percentile population values were derived from four small European studies in pregnant women from the late 1970’s to the 1980’s [7–10]. These studies examined a total of 427 pregnant women between the four studies. The American College of Obstetricians and Gynecologists has also supported this definition of anemia in pregnancy [11]. To date, there have been no studies to determine if they are valid in a modern United States pregnant population.

The normal hematologic changes of pregnancy include an expansion of plasma volume that exceeds the pregnancy-related increase in red blood cell mass which creates a “physiologic anemic state.” This physiologic anemia predisposes women to lower hemoglobin and hematocrit values during pregnancy. Iron deficiency affects 30–38% of women of childbearing age [12]. Several factors can affect hematocrit values in pregnant women, such as short interval pregnancy, preeclampsia, race, socioeconomic status, and pre-existing maternal comorbidities. We sought to define anemia in normal, uncomplicated pregnancies. We gathered data from two cohorts of pregnant women: one cohort at a public county hospital and another at a private hospital and compared these cohorts to identify if there was a difference between the fifth percentiles. We hypothesized that there would be no statistical or clinical difference between the cohorts, and thus combined a definition of anemia in pregnancy could be established.

## Materials and methods

To evaluate a time-independent, representative normal, uncomplicated cohort, the first 250 women in 2011, 2013, 2015 and 2018 to deliver at a large inner-city county hospital (Parkland Health and Hospital Systems) and a private University-Practice hospital (Clements University Hospital) that met inclusion criteria were studied for a total of 2000 women. Women were included if they presented to prenatal care before 20 weeks of gestation. Exclusion criteria included preterm birth (less than 37-weeks gestation), preeclampsia, hypertension, diabetes, short interval pregnancy (less than 18 months from delivery to conception), multiple gestation, placental abruption, pyelonephritis during the pregnancy, maternal sickle cell disease, maternal hemoglobinopathies, placenta previa, placenta accreta spectrum, fetal demise, and patients requiring parenteral iron infusions during the antepartum period. Gestational age at presentation to care and delivery were obtained on all women. All women had iron fortification with ferrous sulfate 325 mg daily recommended throughout pregnancy. Presentation to care hemoglobin/hematocrit, and pre-delivery values were obtained. If hemoglobin (g/dL) levels were not available, they were derived from the hematocrit levels drawn [13, 14]. The fifth percentile for both hemoglobin and hematocrit was determined from the cohorts and the fifth percentile was determined for presentation to care, first trimester, second trimester, and pre-delivery. The cohorts were compared in demographics, hemoglobin/hematocrit values, and their fifth percentiles. The primary outcome was the difference between fifth percentile values at each antepartum epoch between the public and private hospital cohorts. This study was deemed exempt by the Institutional Review Board (IRB) at the University of Texas Southwestern Medical Center (STU 2020–0200) and the IRB waived the requirement for informed consent. The data set was not fully anonymized before it was accessed but was anonymized before data analysis.

After comparison of the cohorts was completed, the two cohorts were combined to create a diverse patient mixed cohort to use for the definition of anemia in normal, uncomplicated pregnancies. The fifth percentile of the combined cohort (n = 2000) as a whole was calculated for each antepartum epoch.

### Statistical analysis

Maternal demographics were compared using the student’s t-test or chi-squared test where appropriate. Fifth percentile values of each antepartum epoch were compared between the cohorts by estimating the fifth percentiles through the empirical distribution (ordered data points) by locating the first point in the ordered distribution which is 5% of the way through the distribution. Estimates of the difference in the fifth percentile between the public and private hospital cohorts were estimated using quantile regression with an indicator variable of hospital location. The estimate of the coefficient was then the estimate of the fifth percentile. Confidence intervals were presented using the Student’s t-distribution for this estimate. The null hypothesis of the test is that the coefficient of the indicator variable is equal to zero. This was then evaluated with the student’s t-test.

## Results

**Fig 1:**
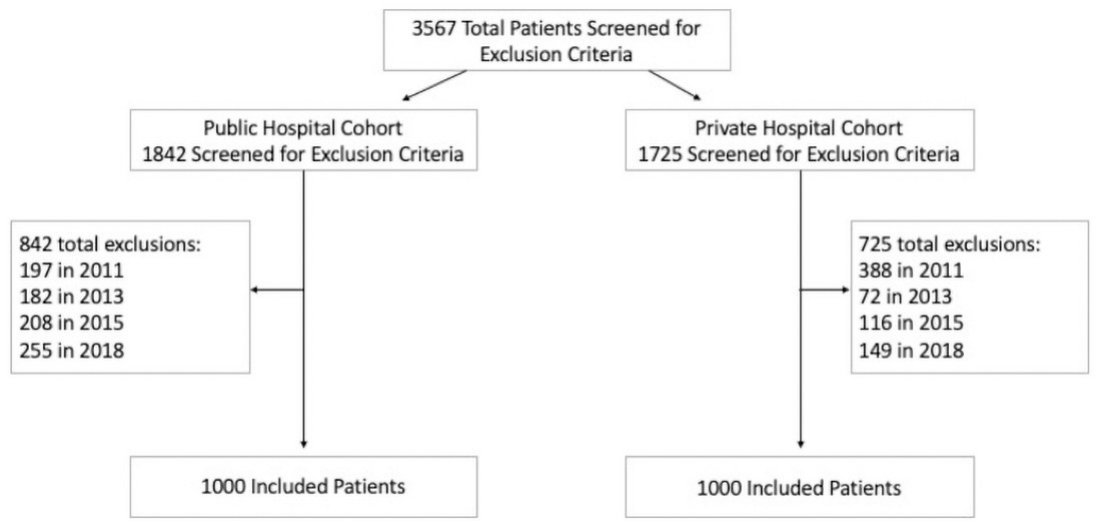
Flow diagram of anemia cohort distribution.

Of the 2000 women, there were 1000 from the public and 1000 from the private hospital. Each cohort of 1000 patients were comprised of the first 250 patients to deliver at each institution in 2011, 2013, 2015, and 2018 that met criteria for inclusion in order to identify women without complications. To identify a normal, uncomplicated cohort of 1000 women in each cohort, 3567 women were screened with 842 excluded in the public hospital cohort and 725 in the private hospital cohort, as per the exclusion criteria (Fig 1). In the public cohort this included 197 women in 2011, 182 women in 2013, 208 women in 2015, and 255 women in 2018 in the public cohort. The private cohort excluded 388 women in 2011, 72 women in 2013, 116 women in 2015 and 149 women in 2018. Maternal demographics and outcomes of each normal, uncomplicated cohort are depicted and compared in Table 1. Those patients in the private hospital cohort were significantly older, more likely to be nulliparous, and had a higher percentage of White women when compared to the public hospital cohort, which had a higher percentage of Hispanic women.

**Table 1:** Maternal demographics and obstetric outcomes.

Characteristic	Public Hospital Cohort (n = 1000)	Private Hospital Cohort (n = 1000)	p-value
Age (years)	27.4±6.2	29.6±5.6	<0.001
Race/Ethnicity			<0.001
Hispanic	850 (85)	121 (12)	
Black	99 (10)	196 (20)	
White	21 (2)	405 (40)	
Other	30 (3)	161 (16)	
Unknown	0 (0)	117 (12)	
Nulliparous	322 (32)	509 (51)	<0.001
Gestational Age at Presentation to Care (weeks)	10.4±3.7	10.8±3.4	0.01
Delivery mode			0.46
Vaginal Delivery	692 (69)	707 (71)	
Cesarean Section	308 (31)	293 (29)	

Data expressed as n (%) or mean ± standard deviation as appropriate. Student’s t-test and Chi-squared tests used where appropriate.

The hematologic indices in each cohort and antepartum epoch are presented and compared in Table 2. Seven hundred and seventy-seven patients presented in the first trimester (less than 14 weeks gestation) in the public hospital cohort, with 785 in the private cohort. When compared by student’s t-test, the hemoglobin mean in each antepartum epoch and the hematocrit mean in the second trimester and third trimester/predelivery were statistically different between the cohorts. The estimates of the fifth percentiles of each antepartum epoch are compared between the public hospital cohort and the private hospital cohort in Table 3. The pre-delivery hemoglobin and hematocrit fifth percentile estimates were statistically different between the cohorts.

**Table 2:** Average hemoglobin/hematocrit per antepartum epoch.

Antepartum Epoch	Public Hospital Cohort (n = 1000)	Private Hospital Cohort (n = 1000)	p-value
Presentation to Care	**N = 1000**	**N = 1000**	
Hemoglobin (g/dL)	12.3±0.9	12.4±1.1	0.03
Hematocrit (%)	36.9±2.7	37.0±3.0	0.43
First Trimester	**N = 777**	**N = 785**	
Hemoglobin g/dL)	12.4±0.9	12.6±1.0	<0.001
Hematocrit (%)	37.4±2.6	37.3±2.9	0.47
Second Trimester	**N = 223**	**N = 215**	
Hemoglobin (g/dL)	11.8±0.9	12.1±1.1	0.002
Hematocrit (%)	35.3±2.7	35.9±3.1	0.03
Pre-Delivery	**N = 1000**	**N = 1000**	
Hemoglobin (g/dL)	12.1±1.1	11.9±1.3	<0.001
Hematocrit (%)	36.2±3.2	35.9±3.4	0.04

Data expressed as mean ± standard deviation.

**Table 3:** Comparison of fifth percentile estimates between cohorts by antepartum epoch.

Antepartum Epoch	Public Hospital Cohort Fifth Percentile Estimate	Private Hospital Cohort Fifth Percentile Estimate	p-value
Presentation to Care	**N = 1000**	**N = 1000**	
Hemoglobin (g/dL)	10.7 (10.6, 10.9)	10.7 (10.6, 10.9)	0.78
Hematocrit (%)	32.2 (31.8, 32.7)	32.2 (31.4, 32.4)	1.00
First Trimester	**N = 777**	**N = 785**	
Hemoglobin (g/dL)	11.0 (10.9, 11.1)	11.0 (10.8, 11.1)	1.00
Hematocrit (%)	33.0 (32.7, 33.4)	32.5 (32.2, 33.0)	0.09
Second Trimester	**N = 223**	**N = 215**	
Hemoglobin (g/dL)	10.2 (9.7, 10.5)	10.4 (9.6, 10.6)	0.55
Hematocrit (%)	30.6 (29.1, 31.4)	30.8 (28.7, 31.6)	0.83
Pre-Delivery/Third Trimester	**N = 1000**	**N = 1000**	
Hemoglobin (g/dL)	10.2 (10.0, 10.4)	9.6 (9.3, 9.8)	<0.001
Hematocrit (%)	30.7 (30.1, 31.1)	29.9 (29.3, 30.4)	0.02

Data presented as Fifth Percentile Estimate (95% Confidence Interval).

The combined cohort of normal, uncomplicated pregnancies fifth percentiles of each antepartum epoch are detailed in Table 4, which can be used to define anemia at each timepoint in pregnancy.

**Table 4:** Anemia defined, fifth percentile values of a normal, uncomplicated population.

Antepartum Epoch	Hemoglobin (g/dL)	Hematocrit (%)
Presentation to Care	10.7	32.1
First Trimester	11.0	32.8
Second Trimester	10.3	30.6
Pre-Delivery/Third Trimester	10.0	30.2

## Conclusions

By comparing the hematologic indices of normal, uncomplicated diverse populations of obstetric patients at each antepartum epoch, we demonstrate that the definition of maternal anemia is consistent. Using this combined uncomplicated, normal pregnancy cohort, we identified normal fifth percentile cutoff values to define anemia in pregnancy. The fifth percentile hematocrit and hemoglobin cutoff values of 10.7 g/dL or 32.1% at presentation to care, 11.0 g/dL or 32.8% in the first trimester, 10.3 g/dL or 30.6% in the second trimester, and 10.0 g/dL or 30.2% pre-delivery can be used for normal, uncomplicated pregnancies.

The European studies from the 1970’s and 1980’s used iron fortified pregnant women to obtain their mean and fifth percentile hemoglobin and hematocrits [6–8, 15]. In turn, the CDC adopted these measures as there were no comparable population-based studies in the United States for normal pregnant women. Our pre-delivery hematocrit in term pregnancies of 30.2% in our selected “normal” population is less than the recognized 33% from the Centers for Disease Control. However, the United States population is different and more diverse than that of Scandinavia, and there have been significant changes in obstetric and medical care within the past 35 years.

In our data set, there were statistical differences between the hemoglobin mean in each antepartum epoch and the hematocrit mean in the second trimester and third trimester/predelivery between the cohorts. However, it could be argued that these differences are clinically irrelevant as these differences are within the standard of error.

Anemia can have many different causes and contributing factors to its development and pathophysiology. To be able to define cutoff values, it is critical to use a normal, healthy and uncomplicated obstetric population. By defining anemia in pregnancy, we are able to identify women at risk of severe maternal morbidity and mortality and circumvent the need for blood transfusion postpartum. Furthermore, this will translate into the timing of interventions such as iron fortification and other measures to increase hematocrit in pregnancy prior to delivery, such as parenteral iron or erythropoietin derivatives [16].

Determining women at the highest risk for blood transfusion, a marker for severe maternal morbidity, is imperative when determining when to intervene in the events surrounding delivery. Defining a hemoglobin cutoff of 10.0 g/dL and hemoglobin cutoff of 30.2% at delivery to be the fifth percentile cutoff values will assist labor and delivery clinicians in determining which patients may be at highest risk for a blood transfusion or severe maternal morbidity surrounding delivery and allow them to prepare by alerting the blood bank or having postpartum hemorrhage treatments readily available at delivery.

This study determines fifth percentile values to define anemia from a modern, United States pregnant cohort. Future studies can focus on defining anemia by maternal outcomes, specifically Severe Maternal Morbidity outcomes and determine if patients who had poor outcomes had differing values than fifth percentile values from a normal, uncomplicated population. Future studies also need to focus on interventions that can prevent SMM in anemic populations prior to delivery.

A strength of our study is that we provide data from a modern United States cohort to define normative values for anemia. Current recommendations are based on small studies that were performed in European countries over 35 years ago. In addition, consistent with studies defining anemia in non-pregnant individuals, we used a normal, healthy population, controlling for obstetric and medical complications that could impact the hematologic values at delivery, which has not been previously described.

One of the main limitations of our study were that our cohort was representative of a majority white-Hispanic population. Only a minority of our combined population (n = 295 or 15%) was African American, a population in which anemia in pregnancy is prevalent. There is support, however, for equivalent iron stores (higher ferritin levels in African American men and women) between Caucasian and African American women [17, 18]. At our public hospital system, we primarily utilize hematocrits because of the nature of our hospital and prenatal care clinic system. Hematocrits, not complete blood counts, are often obtained in our prenatal clinicals and labor and delivery area due to this reason. However, hematocrit and hemoglobin are equivalent measures in most patients, and the equivalent values can be estimated from each other with clinical accuracy [13, 14]. It is also acknowledged that some women in our overall cohort may have underlying iron deficiency anemia or an unknown diagnosis of thalassemia that may skew the results. This could not be clarified in our data set as not all women had iron, ferritin, or mean corpuscular volume drawn during their routine pregnancy laboratory studies. Finally, while iron fortification was recommended for all women, compliance remains a limitation. we were not able to evaluate if each patient was compliant with the recommendation.

These data provide guidance for practitioners defining anemia in a United States population as hematocrit and hemoglobin values of 32.1% or 10.7 g/dL at presentation to care, 32.8% or 11.0 g/dL in the first trimester, 30.6% or 10.3 g/dL second trimester, and 30.2% or 10.0 g/dL pre-delivery. These data will be essential for risk stratification for postpartum hemorrhage, blood transfusion, and understanding severe maternal mortality indicators. With a standard, contemporary definition, an approach to mitigate anemia at delivery and improve pregnancy outcomes is possible.

## Supporting information

S1 Data (XLSX)
